# Venous sinus thrombosis during COVID-19 infection in pregnancy: a case report

**DOI:** 10.1590/1516-3180.2020.0659.R1.08122020

**Published:** 2021-02-15

**Authors:** Zahide Betül Gunduz

**Affiliations:** I MD, PhD. Neurologist and Assistant Professor, Department of Neurology, University of Health Sciences, Konya Education and Research Hospital, Konya, Turkey.

**Keywords:** COVID-19 [supplementary concept], Pregnancy, Headache, Female, Venous sinus thrombosis, Hypercoagulability, Infectious disease

## Abstract

**BACKGROUND:**

Although it is known that the new coronavirus disease (COVID-19), which was first seen in Wuhan, China, in December 2019 and has affected the whole world, mainly targets the respiratory tract, cases of this disease with a wide clinical spectrum are emerging as information is shared.

**CASE REPORT:**

We present the case of a pregnant woman who was diagnosed with venous sinus thrombosis after she developed headache and hemiparesis. Polymerase chain reaction (PCR) positivity lasted for two weeks after COVID-19 had been diagnosed.

**CONCLUSIONS:**

In patients with suspected COVID-19, especially in the presence of causes of hypercoagu- lability and presence of atypical features, venous sinus thrombosis needs to be kept in mind in making the differential diagnosis.

## INTRODUCTION

The effects of COVID-19 are not limited to the lungs alone. After the virus enters the body, it causes various symptoms through viremia.^1^ COVID-19 has been implicated in occurrences of cardiovascular and thromboembolic complications due to systemic inflammation and coagulopathy, in the light of the increasing amount of data that has become available over time.^2^^,^^3^^,^^4^^,^^5^^,^^6^^,^^7^^,^^8^ On the other hand, some published papers have argued that the signs and symptoms of severe COVID-19 infection are more similar to the pathophysiology and phenotype of complement-mediated thrombotic microangiopathy (TMA), rather than to sepsis-induced coagulopathy or diffuse intravascular coagulation (DIC).^8^ It has been suggested that COVID-19 predisposes patients to thrombotic pathological conditions in both the venous and the arterial circulation due to inflammation, platelet activation, endothelial dysfunction and stasis.^4^ Nevertheless, cases of neurological and cardiac involvement in COVID-positive patients with TMA have also been reported.^9^

While the mechanism for the susceptibility to thrombosis that has been seen among COVID-19 patients continues to be debated, we wanted through this report to share information regarding the common venous thrombus of the central nervous system that emerged during the subclinical course of COVID-19 in a pregnant patient, which caused rapid parenchymal infarction.

## CASE REPORT

A 22-year-old patient who was 35 weeks pregnant was evaluated in the emergency department with a complaint of right-sided weakness. The COVID-19 polymerase chain reaction test was performed and was found to be positive. However, she did not have fever or respiratory distress and then was followed up at home without medication.

The patient started to have throbbing headaches that did not respond to analgesic treatment (paracetamol 1000 mg/day) for four days. The intensity of her headaches gradually increased, such that she was being awakened from sleep, and this condition was accompanied by nausea and vomiting.

After this four-day period, she again felt weakness on her right side when she woke up in the morning. Twelve hours later, she went back to the emergency department because her weakness was increasing. At the emergency department, the patient was found to be normotensive, conscious, cooperative and oriented in a neurological examination. No fundus examination was performed, given that she was COVID-positive. Examinations on the patient’s visual field and vision showed normal results. Other cranial nerve examinations were normal. Her muscle strength ratio was 3/5 in the upper right extremity, 2/5 in the lower right extremity and 5/5 in the upper and lower left extremities. The foot sole skin reflex of the right lower extremity consisted of an extensor response. She presented decreased speech fluency and had difficulty in word finding, which were diagnosed as mild motor aphasia. Laboratory tests revealed high levels of fibrinogen (899 g/l; normal is 180-400) and D-dimer (6.38 mg/l; normal is 0-2). It was noted that the patient had also had high levels of fibrinogen (665 g/l) and D-dimer (2.2 mg/l) in examinations performed 10 days previously.

**Figure 1.**
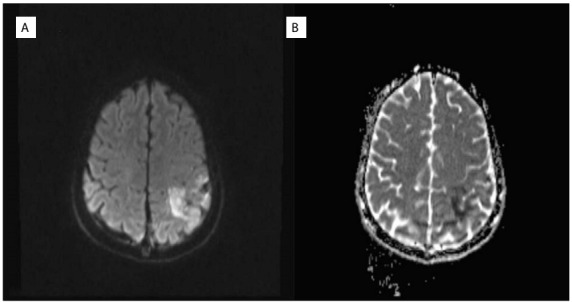
a) Diffusion magnetic resonance imaging; b) Apparent diffusion coefficient.

**Figure 2.**
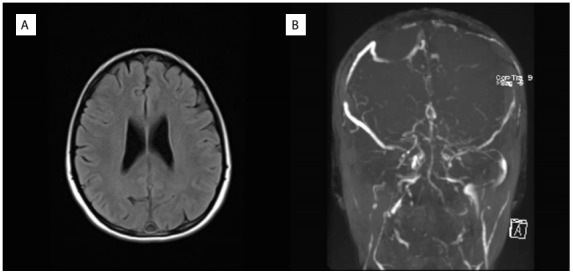
a) Cranial magnetic resonance imaging; b) Magnetic resonance imaging venography.

Diffusion magnetic resonance imaging (MRI) showed cortical diffusion restriction in the left parietal region (Figure 1a) and a hypointense response in the apparent diffusion coefficient (ADC) (Figure 1b). The result from the diffusion MRI was suggestive of venous sinus thrombosis. Widespread loss of flow in the superior sagittal sinus and right transverse sinus, suggesting partial venous thrombosis in the left transverse sinus, was observed in brain magnetic resonance imaging (Figure 2a) and magnetic resonance venous angiography (Figure 2b). The brain MRI and magnetic resonance venography confirmed the diagnosis of venous sinus thrombosis. Thrombosis was not investigated in other parts of the body.

The polymerase chain reaction was repeated and the result was again positive. The patient was then hospitalized with the diagnoses of COVID-19 infection and venous sinus thrombosis. Other genetic, hematological and rheumatological examinations were planned, in order to investigate the etiology of her condition. Anticoagulant treatment (low molecular weight heparin) was started after the patient had been found to present a low platelet count (107,000/mm^3^), through evaluation of a peripheral blood smear.

Diffuse contractions were observed in a non-stress test (NST), and tocolysis was started, consisting of nifedipine and betamethasone treatment. However, the patient’s labor could not be stopped after 18 hours of hospitalization. She was then admitted for an emergency cesarean section because the intracranial pressure was increasing. A healthy baby was delivered.

**Figure 3.**
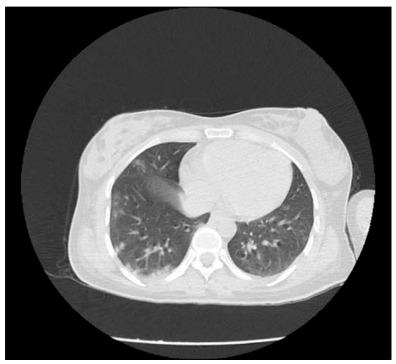
Thoracic computed tomography.

Subsequently, the patient’s postpartum headache complaints decreased and her speech became fluent without any change in muscle strength deficit. Her thrombocyte counts decreased to 67,000/mm^3^. Anticoagulant therapy was continued, with peripheral smear follow-ups. Thoracic computed tomography was performed on the patient, who did not present respiratory distress, and the findings were compatible with COVID-19 pneumonia after birth (Figure 3). There was an increase in infection parameters, and the patient was started on hydroxychloroquine and ceftriaxone treatment.

In the examinations performed to ascertain risk factors, the patient was found to be positive for antinuclear antibodies (ANA) and showed prothrombin heterozygous mutation. The patient was negative for anti-cardiolipin antibodies and anti-double stranded DNA (dsDNA), and tests on lupus anticoagulant, homocysteine, protein C, protein S and antithrombin 3 showed results within normal limits.

On the third day following the birth, the patient’s headache complaint became completely resolved, her thrombocyte counts stopped decreasing and her thrombocytopenia improved over the subsequent days. The infection parameters regressed. Partial regression of the lesions was observed on control thoracic computed tomography. The patient’s general condition stabilized and she was discharged on the 10th day of hospitalization, with muscle strength 4-5/5 on the right side, which was mobilized without support. Continuation of low molecular weight heparin (LMWH) treatment was planned, along with neurological and hematological control tests at a polyclinic.

## DISCUSSION

It is known that the new coronavirus disease (COVID-19), which was first seen in Wuhan, China, in December 2019 and has affected the whole world, mainly targets the respiratory tract. Cases of this disease with a wide clinical spectrum are emerging as information is shared.

Although the mechanism for thrombotic events in the course of COVID-19 remains unclear, it is known that there is a tendency for such events to occur within the course of this disease. Pulmonary embolism was shown to be the cause of death in an autopsy series.^6^ The etiology of embolic events is generally multifactorial: it is accepted that these events are triggered by environmental factors on the basis of genetic predisposition. Nonetheless, no reports on underlying genetic or other acquired causes in cases of COVID-19-positive pulmonary embolism have yet been published in the literature.

An increasing number of case reports and series on COVID-19-positive patients are describing a wide variety of neurological symptoms. Encephalopathy has been reported in a total of 93 patients, including 16 (7%) out of 214 hospitalized patients with COVID-19 in Wuhan, China, and 40 (69%) out of 58 patients in intensive care with COVID-19 in France. To date, encephalitis has been described in eight patients and Guillain-Barré syndrome in 19 patients. SARS-CoV-2 has been detected in the cerebrospinal fluid (CSF) of some patients. Anosmia and agnosia are common and may occur in the absence of other clinical features. Unexpectedly, acute cerebrovascular disease has also emerged as an important complication: stroke was reported in 2-6% of patients hospitalized with COVID-19 in a cohort study.^10^

Li et al. stated that in a retrospective study in which 219 COVID-19 positive patients were screened, 10 of these patients (4.6%) were ischemic and one of them presented hemorrhagic cerebrovascular disease (0.5%), after an average of 10 days after the onset of COVID-19. They pointed out that the mean age of these patients was greater and their cardiovascular and cerebrovascular risk factors were more severe.^11^

COVID-19 is thought to predispose patients to thrombotic pathological conditions in both venous and arterial circulation due to inflammation, platelet activation, endothelial dysfunction and stasis.^4^ The initial signs of coagulopathy due to COVID-19 have been found to be marked increases in fibrin/fibrinogen-degradation products and D-dimer levels. It was observed that our patient had high levels of fibrinogen and D-dimer from the time when she was diagnosed with COVID-19 to the time when she was diagnosed with venous sinus thrombosis.

In the early stages of the disease, abnormalities in prothrombin time, partial thromboplastin time and platelet count are uncommon.^5^ Detection of deep vein thrombosis (58%) as the autopsy finding among more than half of the 12 patients who died of COVID-19, and pulmonary embolism as the cause of death among one third of the patients, has emphasized the importance of not ignoring the tendency towards occurrences of thrombosis in the course of this disease. Hence, anticoagulants should be included during treatment planning.^6^ Analysis on the data on 184 patients with COVID-19 infection who were monitored in an intensive care unit showed that 31% of them had thrombotic complications. Thus, prophylaxis for thrombosis was strongly recommended for patients hospitalized with this diagnosis.^7^

On the other hand, some published papers have argued that the signs and symptoms of severe COVID-19 infection are more similar to the pathophysiology and phenotype of complement-mediated thrombotic microangiopathy (TMA), rather than to sepsis-induced coagulopathy or diffuse intravascular coagulation (DIC).^8^ Thrombotic microangiopathy is characterized by organ damage such as microangiopathic hemolytic anemia, thrombocytopenia, and neurological, renal and cardiac dysfunction. Thrombocytopenia and neurological deficits were also observed in our patient. In another study, anemia, increased lactate dehydrogenase (LDH), thrombocytopenia and organ damage (neurological in all patients and cardiac in one) were explained by thrombotic microangiopathy in three patients with a diagnosis of COVID-19.^9^

Cerebral venous thrombus differs significantly from arterial infarctions in terms of risk factors. Hypercoagulability is an important risk factor and an important cause of stroke in young people. Women are affected three times more often than men. The most common symptoms are headache, seizures and focal neurological deficits. The diagnosis can be confirmed by magnetic resonance imaging, computed tomography-venography or catheter angiography.

The primary treatment for venous sinus thrombosis is anticoagulation, based on the limited evidence from randomized trials. Although a small series of cases has indicated that endovascular therapy may be promising, these data require confirmation through a randomized trial. Decompressive surgery can be lifesaving for patients at risk of herniation. The prognosis is generally better than that for arterial stroke.^12^ Although venous sinus thrombosis was previously considered to be a life-threatening condition, it is known that the mortality rate in these cases declines over time. Moreover, increased clinical awareness, development of neuroimaging techniques and improvement in therapeutic management have provided better prognoses through enabling earlier diagnosis and identification of less severe cases.^13^

Pregnancy and the puerperium are common causes of transient prothrombotic conditions. About 2% of pregnancy-related strokes can be attributed to venous sinus thrombosis. In the puerperium, the rate of venous sinus thrombosis is 12 cases per 100,000 births. This venous rate in the puerperal period is only slightly lower than that of arterial stroke. Women are at risk of venous thromboembolic events during pregnancy and for up to six to eight weeks after delivery. Most cases of pregnancy-related venous sinus thrombosis are seen in the third trimester or, more often, in the puerperium, when the body prepares for delivery through hypercoagulation. In a paper published in Canada, it was reported that frequency of venous sinus thrombosis in the postpartum period is much higher than during pregnancy. In the puerperium period, the presence of infection and use of instrumental delivery or cesarean section increase the risk of venous sinus thrombosis. During pregnancy, it is known that the risk of venous sinus thrombosis increases in the presence of hypertension, infections and excessive vomiting, and as the maternal age increases.^14^ The European Academy of Neurology has recommended that treatment for acute venous sinus thrombosis should start with oral anticoagulant therapy (vitamin K antagonists) for 3-12 months, according to risk factors.^15^

Another risk factor with a relationship to venous sinus thrombosis that is clearly known is inflammation. Venous sinus thrombosis is associated with systemic inflammatory conditions such as Behçet’s disease and inflammatory bowel disease, in addition to infections such as otitis, mastoiditis, sinusitis, dental infections and skin abscesses in neighboring tissues and meningitis.^13^ In the anamnesis and examination of our patient, no finding suggesting adjacent tissue infection or Behçet’s or inflammatory bowel disease was found.

Antinuclear antibody positivity can be seen in autoimmune diseases, especially systemic lupus erythematosus, but it is not a laboratory test specific to autoimmune diseases. Since antinuclear antibody positivity can be observed in acute or chronic infectious processes,^16^ it was planned that our patient would undergo this examination after discharge. The anamnesis of our patient was negative for rheumatological diseases.

The etiology of venous sinus thrombosis can be explained in terms of the classical Virchow triad, i.e. blood flow stasis, vessel wall changes and changes in blood content. We believe that the combination of pregnancy and systemic inflammation due to COVID-19 caused thrombosis in our patient, on the basis of genetic prothrombin heterozygous mutation.

One of the clinical manifestations of COVID-19 is nonspecific headache, as is also frequently observed during other viral infections. However, this symptom can often be mild enough to lag behind other clinical findings. If there is no visual impairment, focal neurological deficit or seizure, venous sinus thrombosis can be neglected in the differential diagnosis. Our patient was diagnosed not after occurrences of headache and nausea-vomiting, but after admission to the hospital because of the accompanying symptoms of right hemiparesis. In our case, like what has been described in the literature,^11^ the central nervous system event started on the ninth day after COVID-19 infection began, and a stroke occurred on the 13^th^ day. Thus, within four days, the rapid clinical progression resulted in parenchymal ischemia. The neurological clinical findings rapidly improved in parallel with the end of pregnancy, start of administration of low molecular weight heparin and decrease in infection parameters.

Reports correlating COVID-19, headache and pregnancy are very rare (Table 1).

**Table 1.** Search of the literature in medical databases for case reports on COVID-19, pregnancy and headache on November 6, 2020

Database	Search strategies	Papers found	Papers related (to pregnancy, headache and COVID-19)	Etiology	Main neurological symptom
MEDLINE (via PubMed)	COVID-19 [MESH] Pregnancy [MESH] Headache [MESH] Case Report [ptyp]	2	2	Spinal anesthesia 1 Pituitary apoplexy 1	Headache
Cochrane	COVID-19 [MESH] Pregnancy [MESH] Headache [MESH] Case Report [ptyp]	0	0	0	0
Embase	COVID-19 [MESH] Pregnancy [MESH] Headache [MESH] Case Report [ptyp]	0	0	0	0

## CONCLUSION

Headache is one of the common symptoms of COVID-19. In the presence of other risk factors accompanying COVID-19, the risk of thromboembolic events increases significantly. Among patients with suspected COVID-19, considering venous sinus thrombosis in the differential diagnosis may be life-saving, through enabling early diagnosis and treatment. This is especially so in the presence of causes of hypercoagulability such as pregnancy, malignancy and presence of atypical features like analgesic unresponsiveness, awakening from sleep, visual impairment, neurological deficits or seizures.
